# High pregnancy rates in humpback whales (*Megaptera novaeangliae*) around the Western Antarctic Peninsula, evidence of a rapidly growing population

**DOI:** 10.1098/rsos.180017

**Published:** 2018-05-02

**Authors:** Logan J. Pallin, C. Scott Baker, Debbie Steel, Nicholas M. Kellar, Jooke Robbins, David W. Johnston, Doug P. Nowacek, Andrew J. Read, Ari S. Friedlaender

**Affiliations:** 1 Fisheries and Wildlife Department, Marine Mammal Institute, Hatfield Marine Science Center, Oregon State University, 2030 SE Marine Science Drive, Newport, OR 97365, USA; 2 Department of Ecology and Evolutionary Biology, University of California Santa Cruz, Coastal Biology Building, 130 McAllister Way, Santa Cruz, CA 95060, USA; 3 Marine Mammal and Turtle Division, Southwest Fisheries Science Center, National Marine Fisheries Service, National Oceanic and Atmospheric Administration, 8901 La Jolla Shores Drive, La Jolla, CA 92037, USA; 4 Center for Coastal Studies, 5 Holway Avenue, Provincetown, MA 02657, USA; 5 Division of Marine Science and Conservation, Nicholas School of the Environment, Duke University Marine Laboratory, 135 Duke Marine Lab Road, Beaufort, NC 28516, USA; 6 Pratt School of Engineering, Duke University, Durham, NC 27708, USA; 7 Institute for Marine Science and Department of Ecology and Evolutionary Biology, University of California Santa Cruz, 115 McAllister Way, Santa Cruz, CA 95060, USA

**Keywords:** humpback whale, progesterone, pregnancy, biopsy, Antarctica, whaling

## Abstract

Antarctic humpback whales are recovering from near extirpation from commercial whaling. To understand the dynamics of this recovery and establish a baseline to monitor impacts of a rapidly changing environment, we investigated sex ratios and pregnancy rates of females within the Western Antarctic Peninsula (WAP) feeding population. DNA profiling of 577 tissue samples (2010–2016) identified 239 males and 268 females. Blubber progesterone levels indicated 63.5% of the females biopsied were pregnant. This proportion varied significantly across years, from 36% in 2010 to 86% in 2014. A comparison of samples collected in summer versus fall showed significant increases in the proportion of females present (50% to 59%) and pregnant (59% to 72%), consistent with demographic variation in migratory timing. We also found evidence of annual reproduction among females; 54.5% of females accompanied by a calf were pregnant. These high pregnancy rates are consistent with a population recovering from past exploitation, but appear inconsistent with recent estimates of WAP humpback population growth. Thus, our results will help to better understand population growth potential and set a current baseline from which to determine the impact of climate change and variability on fecundity and reproductive rates.

## 1. Introduction

As populations of baleen whales recover from past over-exploitation they are re-occupying ecological roles, particularly on their high latitude feeding grounds from which they may have been functionally absent for many decades. For example, in many areas of the Southern Ocean populations of humpback whales (*Megaptera novaeangliae*) are recolonizing feeding grounds in the Antarctic and sub-Antarctic [1,2]. In some of these areas, whales are encountering environmental conditions very different from those that existed prior to their exploitation. One of these areas, the Western Antarctic Peninsula (WAP), is experiencing some of the fastest rates of regional climate change on Earth. This region has experienced a rise in temperature of nearly 7°C since the 1950s, resulting in the collapse of ice shelves, the retreat of glaciers and the exposure of new terrestrial habitats [3–5]. A decline in seasonal sea ice has been observed along the WAP, resulting in an annual occurrence of sea ice (greater than 15% cover) that is, on average, 80 days shorter than it was four decades ago [5]. This warming is proceeding with a variety of impacts on the ecology of the system [5,6], including regime shifts among sympatric krill predators along the WAP, such as declines in the abundance of Adélie penguins and their replacement by sub-Antarctic gentoo penguins [5].

Like most mysticetes, humpback whales exhibit an annual migration from low-latitude breeding grounds to high-latitude feeding grounds where they exploit high levels of seasonal productivity [7]. The timing of this migration varies as a function of age, sex and reproductive state [8]. During the austral summer and fall, humpback whales feed throughout the Southern Ocean. For management purposes, the International Whaling Commission (IWC) divides this distribution into six feeding areas and includes the WAP within Area I [1,9,10]. The WAP is an important feeding ground for humpback whales (breeding stock G) that winter along the west coast of South and Central America (e.g. Ecuador, Colombia and Panama) and perhaps other populations as well [9,11]. The timing of migration, and therefore the duration of feeding during the austral summer and fall is critical to fuelling the migration of whales to their low latitude breeding grounds [7]. Energetic studies of blue and fin whales have shown that roughly 25% of their annual lipid reserves are used over the course of the annual migration [12]. These energetic demands are even greater for pregnant or lactating whales. Lockyer [12] suggested that lactation and pregnancy will consume roughly 19% of total energy stores in mature female fin whales.

Little is known about the exact timing of migratory movements and residency times of different age and sex classes of humpback whales along the WAP. Our lack of knowledge stems largely from the rapid depletion of humpbacks at the very beginning of Antarctic whaling during the first part of the last century [13], as well as the significant logistical challenges associated with studying whales in polar regions. During this period of whaling, humpback populations were a favoured target because they were relatively easy to catch, abundant in protected bays, and floated when killed [14]. The rapid depletion of these stocks has been well documented [13]. For example, humpbacks comprised the majority of the catch at South Georgia from 1904 to 1910, but by the 1913–14 season this species comprised less than 20% of the whales taken [15]. The same story of unconstrained over-exploitation was repeated in the South Shetlands and along the WAP [14]. As a result, there was little opportunity to study the ecology of humpback whales before they were reduced to remnant populations. An improved understanding of the demography and seasonal patterns of movements of humpback whales in this area would help place current research on their regional density and population dynamics into a broader ecological context.

Identification of the sex of individuals facilitates the study of population structure, behaviour, breeding patterns and social systems [16]. As both iconic animals and top predators, understanding such knowledge about humpback whales allows us to better understand how individuals interact and organize themselves within their environment, as well as provide insight as to the health, structure, and function of the marine ecosystem of which they are a part. Insights into the timing of migration for various age and sex classes are available from other populations of humpback whales (east and west Australia) in the Southern Ocean that were exploited later than those along the WAP [17]. In these areas, pregnant female humpback whales were the first to arrive on the feeding grounds, followed by immature animals, resting females and mature males, and lastly, lactating females and their young calves [7]. These observations were generated during the height of commercial whaling, and there have been no attempts to assess these patterns along the WAP until now.

Estimates of pregnancy are important for assessing density dependent effects predicted for populations as they recover and approach pre-exploitation or presumed carrying capacity [18]. Historically, assessment of the reproductive status of cetaceans was obtained from examination of carcasses taken in commercial hunts [19,20]. Since the protection of humpback whales by the IWC in 1966 this source of information has become unavailable for this species [19].

Since protection from hunting, reproductive rates have been estimated for some populations of humpback whales from crude birth rates (% of calves) [21] or by estimating calving rates derived from long-term sighting histories of individual mature females [22–25]. In this approach, estimates of reproductive rates were generated through repeated observations of adult females with and without calves. This approach provides an estimate of the number of recruits entering a population but, without correction, inherently underestimates true fecundity rate, because it does not account for fetal and perinatal mortality. Rates of fetal and perinatal mortality can vary and are potentially important indicators of population health [26].

More recently, biochemical techniques have been developed to detect pregnancy using non-lethal sampling and in some cases making it possible to estimate reproductive rates prior to parturition. For example, pregnancy status has been determined by assessing the concentrations of progesterone in the milk of lactating bottlenose dolphins [27], sex steroids secreted in the urine of killer whales [28], salivary steroids in Hawaiian monk seals [29], blood plasma, salivary, ocular, and vaginal secretions of false killer whales [30], and ultrasound evaluations of dolphins examined during capture–release health assessments [31]. However, such methods are not practical for use with large whales, which cannot be captured or handled. The measurement of progesterone in samples that can be collected in the field, such as faecal material [32], blow [33,34] and skin biopsies [19,20,35] offer a pragmatic alternative for these animals.

Progesterone, often referred to as the hormone of pregnancy, is a lipophilic circulatory steroid hormone produced by the corpus luteum, and is the primary regulator of oestrous cycling and pregnancy [36]. Progesterone's lipophilic properties make biopsy samples of skin and blubber a readily obtainable, non-lethal, analytical matrix for assigning pregnancy in free-swimming cetaceans. This work was pioneered in a large-scale study of short-beaked common dolphin (*Delphinus delphis*), northern right-whale dolphin (*Lissodelphis borealis*) and Pacific white-sided dolphins (*Lagenorhynchus obliquidens*) killed incidentally in the California gill net fishery [20]. The pregnancy status of these female dolphins was determined by physical examination of the carcass, by noting number of corpora, corpus luteum size and/or length of the fetus (if present) [20]. The authors then validated blubber sample endocrine techniques by corroborating their progesterone values with the known reproductive state of the carcass; elevated progesterone concentrations were correlated with pregnant females.

The development of non-lethal tissue sampling techniques [37], and the methods to assess hormone levels from skin–blubber biopsy samples, now provides the capacity to assess demographic rates in wild cetacean populations [19,20,35]. The objective of our study was to assess variation in the sex ratios and pregnancy rates of humpback whales along the WAP, and in doing so, develop a baseline with which to assess the impact of climate change and variability, that affect both available habitat and prey.

## 2. Methods

### 2.1. Biopsy collection

**Figure 1.**
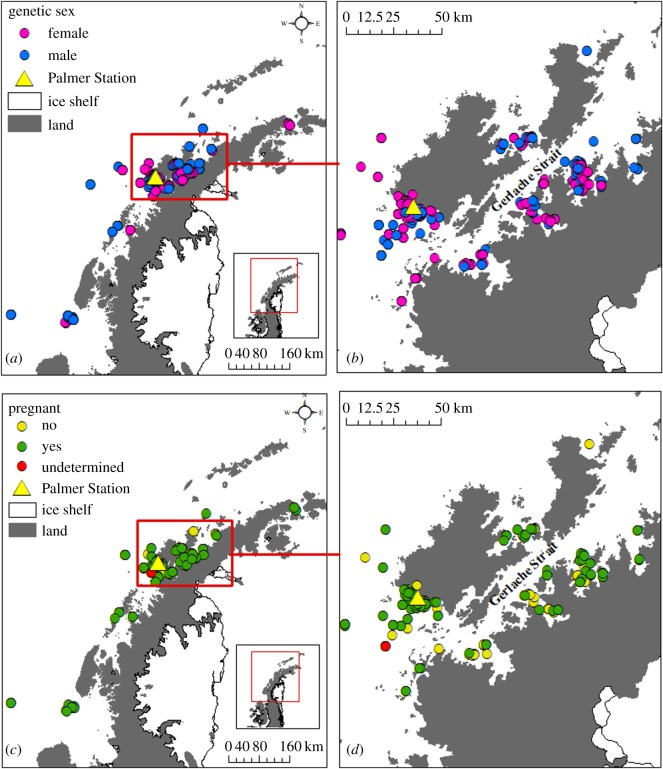
Genetic sex of humpback whales sampled along the Western Antarctic Peninsula (*a*) and in the Gerlache Strait and adjacent bays (*b*) and pregnancy status of female humpback whales sampled along the Western Antarctic Peninsula (*c*) and in the Gerlache Strait and adjacent bays (*d*) during the 2010, 2013–16 field seasons.

We collected skin and blubber samples from humpback whales during the 2010 and 2013–16 austral summer and fall (January–June) field seasons along the WAP using standard biopsy techniques [37]. Samples were collected whenever whales were encountered during prey or visual surveys in an area within approximately 10 nautical miles of Palmer Station, Anvers Island. We also collected samples during the Palmer Long Term Ecological Research (LTER) research cruise in January and February of each year (figure 1). Samples were collected unselectively from all age/sex classes, except young of the year calves, which were excluded. Calves of the year were assumed to have been born the previous breeding season as evident by their small size (less than half of the presumed mother's length) and close association with an adult, presumed to be the mother. Samples were collected using a crossbow with modified bolts and 40 mm tips (CetaDart) from a small vessel at distances of 10–30 m targeting the dorsal and lateral body surfaces near the dorsal fin [37]. Samples were stored frozen whole at −20°C until used for analysis.

### 2.2. DNA profiling

A standard DNA profile, including microsatellite genotypes and sex-specific markers, was used to identify individuals [38]. Total genomic DNA was extracted from the skin–blubber interface using a proteinase K digestion followed by a standard phenol–chloroform extraction [39]. The sex of each sampled whale was identified by amplification of sex-specific markers following the protocol of Gilson *et al.* [40]. We compared results to controls for a known male and female using gel electrophoresis. Sex ratios, depicted as the ratio of males to females (M : F), were calculated for the entire dataset, within years, and across seasons. Each sample was also genotyped using 10 previously published microsatellite loci to determine individual identity and remove potential replicate samples (table 1) [41–45]. Alleles were sized and binned using the software program Genemapper v3.7 (Applied Biosystems). The total number of amplified loci for a given sample was considered as an added quality control threshold. Given the estimated probability of identity for these loci from previous studies [46], we considered that 7 loci were sufficient for individual identification. Samples with fewer than 7 microsatellite loci were re-analysed or excluded. The expected probability of identity (*P*_ID_; the probability that two individuals drawn at random from a population will have the same genotype by chance) for each locus was calculated in GenAlEx v6.5 [47]. Cervus 3.0.7 [48] was used to compute the number of alleles (*K*), observed and expected heterozygosity (*H*_O_ and *H*_E_), deviation from Hardy–Weinberg equilibrium with a Bonferroni correction, and the probability of identity for all individual matches.

**Table 1.** *Megaptera novaeangliae.* Summary of microsatellite loci used for individual identification of humpback whales along the WAP. The number of alleles, observed (*H*_O_) and expected (*H*_E_) heterozygosity, and deviation from Hardy–Weinberg equilibrium were calculated using Cervus 3.0.1. The expected probability of identity (*P*_ID_) of each locus was calculated with the program GenAlEx v6.5.

locus	source	label	[mgCl_2_] mM	size range (bp)	no. of alleles	*H*_E_	*H*_O_	*P*_ID_
Ev14	Valsecchi & Amos [41]	VIC	2.5	125–143	9	0.787	0.748	0.074
Ev37	Valsecchi & Amos [41]	NED	3.5	192–228	18	0.898	0.891	0.019
Ev96	Valsecchi & Amos [41]	FAM	1.5	141–173	15	0.869	0.862	0.030
GATA417	Palsbøll *et al.* [42]	FAM	2.5	143–199	21	0.912	0.891	0.37
GATA28	Palsbøll *et al.* [42]	NED	2.5	187–282	14	0.404	0.402	0.015
GT211	Palsbøll *et al.* [42]	FAM	2.5	100–120	10	0.82	0.82	0.056
GT23	Berube *et al.* [43]	VIC	2.5	101–123	9	0.749	0.712	0.1
GT575	Berube *et al.* [43]	FAM	1.5	137–177	14	0.804	0.787	0.061
rw4–10	Waldick *et al.* [44]	VIC	2.5	190–216	14	0.845	0.824	0.043
rw48	Waldick *et al.* [44]	NED	3	112–120	5	0.724	0.742	0.12

### 2.3. Hormone extraction and quantification

Hormone extraction followed standard methods [20,35]. In brief, a cross-sectional sub-sample (approx. 0.15 g) spanning from the epidermis–blubber interface to the most internal layer of the biopsy was sub-sectioned from female whales. These sub-samples were homogenized separately in 1400 µl of ethanol using an automated, multi-tube homogenizer (Bead Ruptor 12, Omni International). The resulting homogenates were then rinsed with a series of ethanol, 4 : 1 ethanol : acetone, and ethylether washes. Following each rinse, the supernatant was collected. Lastly, the progesterone was separated from the resulting lipid residue using a biphasic acetonitrile–hexane separation. The final hormone pellet was dried down and stored at −20°C until analysis.

Progesterone concentrations were quantified using a progesterone enzyme immunoassay (EIA; Enzo Life Sciences, kit ADI-900-011). Prior to analysis, samples were re-suspended in 1 ml of phosphate buffered saline (pH 7.5) containing 1% bovine serum albumin and vortexed thoroughly. The progesterone EIA kit used in this study has 100% reactivity with progesterone and an assay detection limit between 15 and 500 pg ml^−1^ using the standard curve. Two additional standard dilutions were added to allow for a lower detection limit of the standard curve to 3.81 pg ml^−1^. Samples were run blind and in duplicate. If a sample failed to fall within the detection limit of the assay curve, the samples were re-run at varying dilutions. The reported inter-assay coefficient of variation (COV) and intra-assay COV of the progesterone EIA were in the ranges 2.7–8.3% and 4.9–7.6% respectively. Progesterone concentrations are reported as nanograms of progesterone per gram of blubber (ng g^–1^).

We determined extraction efficiency with every progesterone extraction using samples from a dead, stranded animal of known pregnancy status by spiking subsamples of blubber with 150 ng of progesterone and including these with every extraction [20]. The percentage of progesterone recovered after the extraction was calculated and each sample concentration was adjusted to this efficiency prior to statistical analyses. An extraction efficiency greater than 60% was adequate and is based off the reported range of efficiencies that are seen using these methods [20]. If the efficiency of an extraction set was less than 60%, the sample extracts were discarded and the blubber samples were re-extracted and re-analysed.

A series of serially diluted pooled samples from five humpback whale individuals from the WAP were compared against the EIA kit progesterone standard to determine parallelism [49,50]. The series of serially diluted pooled samples and kit standards were run in duplicate and an analysis of covariance (ANCOVA) was used to determine if the slopes between the two lines were significantly different.

### 2.4. Pregnancy classification

Pregnancy was assigned using the methods developed by Pallin *et al.* [51]. Briefly, we used the progesterone concentrations from a collection of female humpback whales of known pregnancy status, based on annual calving observations, to develop a predictive model. We interpreted the pregnancy state of females sampled along the WAP based on the relationship of their progesterone concentration with the reference levels from known pregnant animals. The model returned the probability that each female was pregnant [51]. If the probability of being pregnant was greater than 99.9%, that female was assumed to be pregnant. If the probability of being pregnant was less than 0.1%, that female was classified as not-pregnant. If a female's probability of being pregnant was between those two bounds, she received an undetermined pregnancy assignment. Lastly, after all probabilities were assigned, we determined the probability of a sample being miss-classified.

### 2.5. Data preparation and statistical analyses

We used a two tailed exact binomial test [52] to test for deviations from a 1 : 1 sex ratio (parity) across the entire dataset, within a given year, and within each season. We separated the austral seasons of summer (January–March) and fall (March–June) using the autumnal equinox on 19 March. Additionally, to avoid re-sample bias in our analyses, we removed all within-year replicates from the inter-annual comparisons; we also removed within season replicates from the same year from the seasonal analyses. In both cases, the most recent sample was retained for the analyses.

We tested for differences in the sex ratios and pregnancy rates across all years and across both seasons by using a *χ*^2^ test of independence. Additionally, we used a Tukey *post hoc* stepwise multiple comparison test to determine if there was a significant difference in pregnancy rates between any two individual years. For all statistical tests, we considered a *p*-value of less than 0.05 to be significant. All values are expressed as mean ± s.d., unless otherwise stated.

## 3. Results

### 3.1. Individual identification and sex

We collected 583 biopsy samples from the waters around the WAP over the course of five field seasons from 2010 to 2016 (figure 1). On average, 9.8 loci were successfully genotyped per individual. The average *P*_ID_ for any given combination of 7 loci ranged from 4.29 × 10^–11^ to 4.83 × 10^–8^, consistent with previous studies. Two samples failed the initial genotype quality control and were re-analysed. Consequently, we considered samples with matching genotypes to be recaptures of the same individual. The DNA profiling was sufficient to identify and determine sex of 507 individual whales from these samples (table 2). In total, we sampled 239 individual males and 268 individual females over the course of the study. Details on annual sampling can be found in table 2. We resampled 54 individuals within the same year (table 3). Three of these individuals were sampled three times (two in 2014, one in 2016) and one individual was sampled four times in 2016. Additionally, we recaptured 11 individuals between years (table 3).

**Table 2.** Sample summary statistics for humpback whales sampled along the WAP (2010, 2013–16) with a known genetic sex. Numbers inside () designate when all replicates have been removed from the sample dataset.

			male		95% CL	female		95% CL	sex ratio	difference to parity
temporal scale	no. samples	no. genotypes	*N*	*%*	*lower–upper*	*N*	*%*	*lower–upper*	*(M : F)*	*exact binomial test*
2010 fall	27	25	8	32.00	14.9–53.5	17	68.00	46.5–85.05	0.47	*p* = 0.108
2010 total	27	25	8	32.00	14.9–53.5	17	68.00	46.5–85.05	0.47	*p* = 0.108
2013 summer	86	79	45	56.96	45.33–68.06	34	43.04	31.94–54.67	1.32	*p* = 0.260
2013 total	86	79	45	56.96	45.33–68.06	34	43.04	31.94–54.67	1.32	*p* = 0.260
2014 summer	109	92	48	52.17	41.5–62.7	44	47.83	37.3–58.5	1.09	*p* = 0.755
2014 fall	27	24	12	50.00	29.12–70.88	12	50.00	29.12–70.88	1	*p* = 1.00
2014 total	136	116 (115)	60 (59)^a^	51.30	41.81–60.73	56	48.70	39.27–58.19	1.05	*p* = 0.852
2015 summer	99	96	43	44.79	34.63–55.29	53	55.21	44.71–65.37	0.81	*p* = 0.358
2015 fall	29	28	10	35.71	18.64–55.93	18	64.29	44.07–81.36	0.56	*p* = 0.185
2015 total	128	124	53	42.74	33.9–51.94	71	57.26	48.06–66.10	0.75	*p* = 0.127
2016 summer	104	95	46	48.42	38.04–58.9	49	51.58	41.1–61.96	0.94	*p* = 0.838
2016 fall	96	83	35	42.17	31.4–53.51	48	57.83	46.49–68.6	0.73	*p* = 0.188
2016 total	200	178 (175)	81 (80)^a^	45.71	38.18–53.40	97 (95)^a^	54.29	46.6–91.82	0.84	*p* = 0.290
total	577	518 (507)	245 (239)	47.14	42.72–51.59	273 (268)	52.86	48.41–57.28	0.89	*p* = 0.213

^a^Denotes where an individual(s) was recaptured across a season.

**Table 3.** Within- and between-year genotype recaptures of humpback whales sampled along the WAP. Recaptures within the same year are presented as male/female. Blue shaded cells indicated male recaptures and pink shaded cells indicate female recaptures.

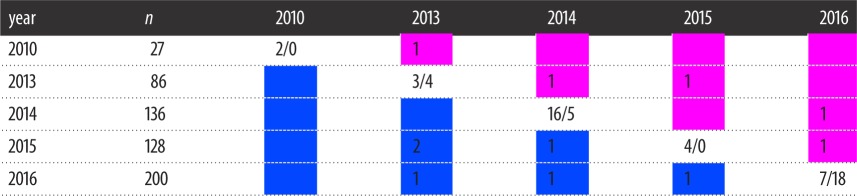

### 3.2. Annual and seasonal variation in sex ratios

Overall, we sampled more females than males (0.89 M : F), but this deviation from parity was not significant *(p* = 0.213, exact binomial test; table 2). Nor did the sex ratio differ significantly from unity in any given year (table 2) and we observed no difference in the sex ratio across all years (*χ*^2^ = 7.256, d.f. = 4, *p* = 0.123; table 2). The sex ratio in summer was very close to parity, 1.01 M : F (182 males, 180 females; exact binomial test, *p* = 0.960). We did, however, observe a significant skew in favour of females in the fall, 0.68 M : F (65 males, 95 females; exact binomial test, *p* = 0.020). This difference in the sex ratio across seasons was significant (*χ*^2^ = 4.146, d.f. = 1, *p* = 0.042).

### 3.3. Validation of humpback progesterone assays

Based on the concentrations observed from the series of spiked controls, our average extraction efficiency was 73.78% ± 0.09 (minimum 61.1%, maximum 95.6%). Additionally, our calculated inter-assay and intra-assay COV from a series of replicated samples was 6.37 and 7.75% respectively. The results from our assay parallelism test between the kit progesterone standard and humpback whale progesterone showed strong parallelism, indicating that the use of the progesterone assay to detect humpback whale progesterone is viable. This result was consistent with previous studies on humpback whales [53]. Specifically, the slopes of the two linear curves were not significantly different (*p* = 0.848; see electronic supplementary material, figure S1).

### 3.4. Pregnancy assignment

**Figure 2.**
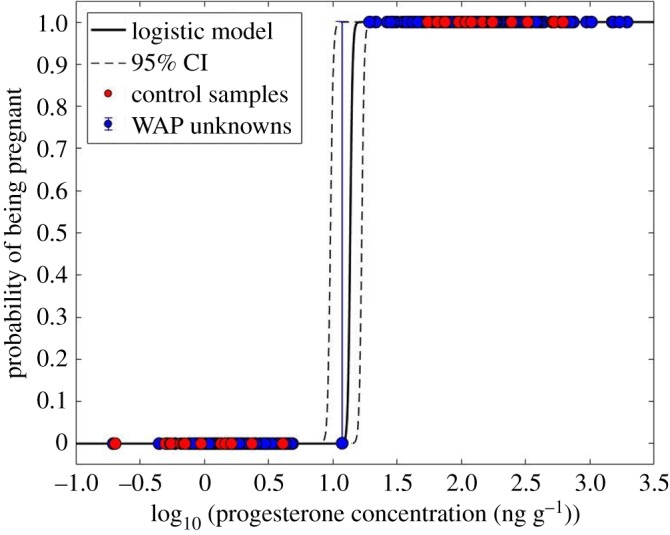
Logistic model used to assign the probability of pregnancy in humpback whales sampled along the WAP. Model was previously described in Pallin *et al.* [51].

We measured progesterone concentrations in 264 samples obtained from 244 individual female humpback whales (figure 1). A small number of samples were excluded from the analysis due to within year re-sampling or insufficient blubber for an extraction. Based on the relationship of their progesterone concentration with the reference levels from known pregnant animals, 89 individuals were assigned as not pregnant (*p* < 0.1% pregnant; blubber progesterone: mean = 2.10 ± 1.13 ng g^–1^; table 4) and 155 were assigned as pregnant (*p* > 99.9%; blubber progesterone: mean = 249.96 ± 281.79 ng g^–1^; table 4). Only a single individual had a probability of pregnancy between 0.1% and 99.9% (blubber progesterone: 11.81 ng g^–1^, probability of pregnancy 0.67%, CI 0.00% to 99.8%; table 4). Out of the 266 samples analysed for progesterone, we estimate that probability of a sample being miss-classified was 3.2 × 10^−6^ (8.6 × 10^−4^/266; CI 4.06 × 10^−7^ to 1.05 × 10^−5^). The within-year replicate samples provided further validation of the assay by demonstrating that re-sampled females continued to fall within the same pregnancy designation. Specifically, 18 of the 19 resampled females showed agreement with pregnancy assignments among all re-samples (mean difference, pregnant = 202.1 ± 394.86 ng g^–1^, not pregnant = 1.45 ± 1.29 ng g^–1^). The one inconsistency occurred in an individual with three samples from the upper flank below the dorsal fin and a sample from the fluke, which lacked a sufficient blubber layer. This sample was not included in any subsequent analyses (table 5). The progesterone concentrations across the two assigned pregnancy states for females sampled along the WAP were distributed in a similar manner to the control samples from the Gulf of Maine described in Pallin *et al.* [51] (figure 2).

**Table 4.** Progesterone concentrations (ng g^−1^) of humpback whales biopsied along the WAP with a pregnancy assignment. Values in () denote the exclusion of within year replicates.

	mean (ng g^–1^)	s.d.	min	max	*N*
not pregnant	2.06 (2.10)	1.12 (1.13)	0.20	4.86	98 (89)
pregnant	254.65 (249.96)	293.94 (281.79)	19.28	1940.52	166 (155)
undetermined	11.81				1
total					264 (244)^a^

^a^Total does not include undetermined individual.

**Table 5.** Summary statistics of pregnancy assignments for female humpback whales sampled along the WAP (2010, 2013–16). Numbers inside () designate when all replicates have been removed from the sample dataset.

			not-pregnant		95% CL	pregnant		95% CL
temporal scale	no. females	no. individuals	*N*	%	lower–upper	*N*	%	lower–upper
2010 fall	11	11	7	63.64	30.79–89.07	4	36.36	10.93–69.21
2010 total	11	11	7	63.64	30.79–89.07	4	36.36	10.93–69.21
2013 summer	35	33	18	54.55	36.35–71.89	15	45.45	28.11–63.65
2013 total	35	33	18	54.55	36.35–71.89	15	45.45	28.11–63.65
2014 summer	41	40	7	17.50	7.34–32.78	33	82.50	67.22–92.66
2014 fall	11	11	0	0.00	0–28.49	11	100.00	71.51–100
2014 total	52	51	7	13.73	5.7–26.26	44	86.27	73.74–94.3
2015 summer	48	48	23	47.92	33.29–62.81	25	52.08	37.19–66.71
2015 fall	16	16	3	18.75	4.05–45.65	13	81.25	54.35–95.95
2015 total	64	64	26	40.63	28.51–53.63	38	59.38	46.37–71.49
2016 summer	44	39	18	46.15	30.09–62.82	21	53.85	37.18–69.91
2016 fall	58	48	14	29.17	16.95–44.06	34	70.83	55.94–83.05
2016 total	102	87 (85)	32 (31)^a^	36.47	26.29–47.62	55 (54)^a^	63.53	52.38–73.71
total	264	246 (244)	90 (89)	36.48	30.43–42.86	156 (155)	63.52	57.14–69.57

^a^Denotes where an individual(s) was recaptured across seasons.

### 3.5. Annual and seasonal variation in pregnancy rates

**Figure 3.**
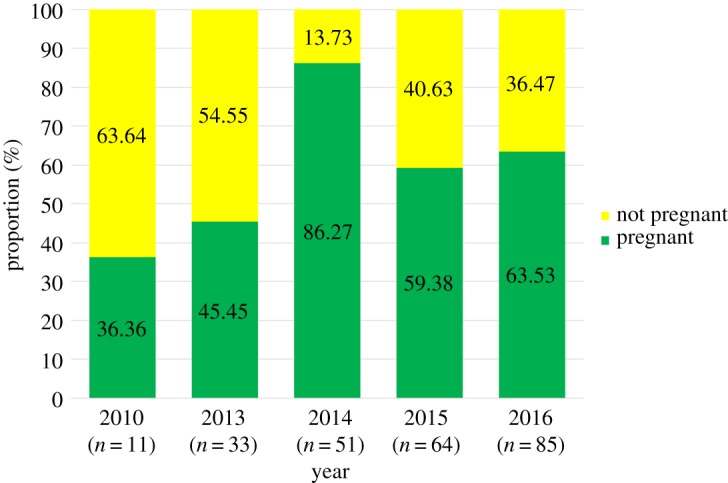
Inter-annual variation in the proportion of assigned pregnant and not pregnant (pregnancy rate) female humpback whales sampled along the WAP based on progesterone concentrations. The overall mean pregnancy rate across all years was 63.5%.

**Figure 4.**
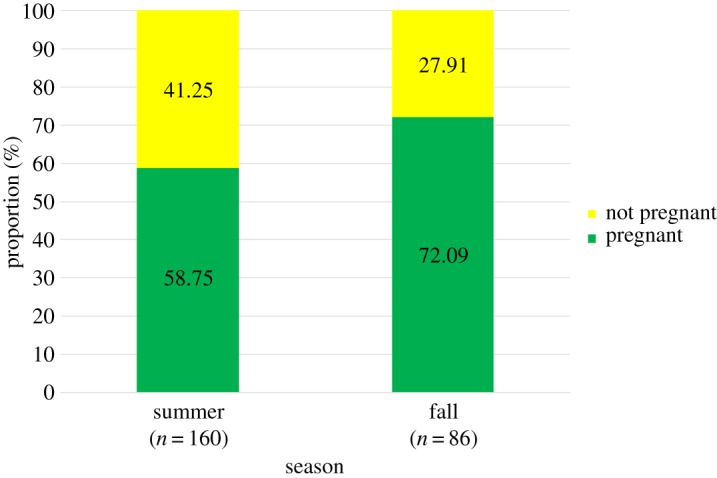
Seasonal variation in the assigned proportion of pregnant and not pregnant (pregnancy rate) female humpback whales sampled along the WAP based on progesterone concentrations.

The mean pregnancy rate for all females across all five years was 63.5%, regardless of the presence of a calf. We observed significant variation in pregnancy rates across years (*χ*^2^ = 20.02, d.f. = 4, *p* = 0.001; figure 3). A *post hoc* multiple comparisons analysis revealed that the pregnancy rate in 2014 was significantly higher than in all other years (2010 *p* = 0.013, 2013 *p* = 0.001, 2015 *p* = 0.02, 2016 *p* = 0.049). Females sampled in the fall had a pregnancy rate of 72.09%, significantly greater (*χ*^2^ = 4.53, d.f. = 1, *p* = 0.001) than that of females sampled in the summer (58.75%; figure 4).

### 3.6. Evidence of annual pregnancy

Of the total of 244 individual females analysed for progesterone, 44 were accompanied by a calf of the year (2010 *n* = 1, 2013 *n* = 4, 2014 *n* = 10, 2015 *n* = 17, 2016 *n* = 12), as verified from field observations. Of these 44, 24 (54.5%; 2010 *n* = 1, 2013 *n* = 1, 2014 *n* = 9, 2015 *n* = 7, 2016 *n* = 6) were pregnant when they were biopsied, indicating the occurrence of annual pregnancy within this population. We also sampled one female in 2015 (Mn15_068H) and 2016 (Mn16_034A-L), that was pregnant in both years.

## 4. Discussion

### 4.1. Variation in sex ratios

The sex ratio of the sampled population was close to unity (0.89 M : F), and we observed no significant differences in sex ratios across years. Our analysis thus supports early observations, derived from catch data, that the sexes mix randomly on the feeding grounds in the Southern Ocean [7,17,54]. Chittleborough [17] reported that males comprised 52.8% of more than 18,000 humpback whales killed in Antarctic Area IV (south of Australia and New Zealand) between 1949 and 1962. Additionally, Matthews [54] reported that 45.4% of the 1057 whales killed at South Georgia were male. Similarly unbiased sex ratios have also been reported from feeding grounds of the North Atlantic [55] and North Pacific [45].

However, we observed a significant bias towards females as the feeding season progressed into the austral fall. This result corroborates prior observations of the temporal segregation of humpback migration in the Southern Hemisphere based on individual demographic status. Specifically, Dawbin [7] reported that during the northbound migration lactating females were the first whales observed to be migrating north to the breeding grounds near Cook Strait, New Zealand. These whales started their migration about one month earlier than the first northbound catches, and were followed by immature whales, mature males, resting females, and lastly, pregnant females [7], resulting in an increased proportion of females on the feeding grounds into the fall.

### 4.2. Variation in pregnancy rates

We observed a pregnancy rate of 63.5% across all years. Interestingly, we also documented evidence of an annual reproductive cycle in which postpartum ovulation and conception occurred in some females. The pregnancy rate of females varied significantly among the 5 years of sampling, ranging from 36 to 86%. This overall rate is higher than reports of the pregnancy rate of female humpbacks taken in Antarctic whaling areas IV and V (mean = 48%) from 1950 to 1956 [53,56]. Our estimate of pregnancy rates is also greater than expected from observed calving rates of 0.37–0.41 calves/year/mature female from Northern Hemisphere populations of humpback whales [57,58], which are also recovering from past exploitation [59–61], but these latter rates are not directly comparable to those presented here, because they include the unknown effects of fetal and perinatal morality.

Calving rates are generated by observing females accompanied by calves that have survived long enough to be observed. Absent sampling biases, the difference between this and pregnancy rates should reflect the rates of fetal loss and perinatal mortality that occur between the dates of pregnancy determination (in this case the biopsy date) and the dates of observations of mothers and calves. In addition, our pregnancy metric reflects the proportion of females that are pregnant, irrespective of maturity state. Typically, calving rates are reported as the fraction of mature females observed with a calf. These distinctions are extremely important when drawing comparisons between our work and other studies.

We observed the highest annual pregnancy rate (86%) in 2014. Similarly high rates of 72% and 82% were documented from catch data in Antarctic whaling areas IV and V from 1950 to 1956 and the Bellingshausen Sea, just west of the WAP, in February of 1957 [56,62]. However, these estimates may be positively biased, as lactating females accompanied by calves were protected by international regulation during this period [56]. Over the course of six years of whaling in Antarctic areas IV and V, pregnancy rates varied from 36% to 72%, similar to the variation we observed over the five years of our study [56].

Observed pregnancy rates along the WAP increased from summer to fall. We believe that pregnant females maximize time spent on the feeding grounds prior to migration. This interpretation is consistent with the bias in favour of females that we observed into the fall, as well as the demographic segregation of migration observed from northbound whales during the whaling era [7,17]. Specifically, there was an increase in the proportion of pregnant females caught in late July and August off Cook Strait, New Zealand [7]. Lockyer [12] suggested that baleen whales must increase their body mass up to 65% to maintain energetic costs for the next year, so pregnant females should stay as long as possible on the feeding grounds to increase their lipid reserves. Lockyer [63] demonstrated that this delay in migration allowed pregnant females to acquire an additional 10–15% of fat reserves than resting females.

### 4.3. Evidence of annual pregnancy

The frequency of post-partum pregnancy and annual calving in humpback whales is poorly understood. We sampled 44 mothers accompanied by calves over the course of our five field seasons, and more than half of these lactating females were pregnant. Chittleborough [64] concluded that female humpback whales are seasonally polyoestrous, with oestrus occurring on the breeding grounds from June to October [54]. There is a general lack of information on the termination of these cycles, but they are believed to occur at conception or when the southward migration begins. Most cycles are characterized by a single ovulation [17,65]. The total number of ovulations over a 2–3 year breeding cycle, however, still remains unclear [66]. Chittleborough [66] documented post-partum ovulation by examining the carcasses of female whales during the commercial whaling era. Specifically, of 19 lactating females he examined at Western Australian whaling stations between 1949 and 1955, eight (42.1%) were both pregnant and lactating [66]. This is similar to the proportion we observed (54.5%) among females sampled along the WAP, almost 60 years later.

There are few other records of annual reproduction in humpback whales in the literature derived from catch records, likely because the IWC prohibited the killing of lactating females accompanied by calves. However, several studies have documented annual reproduction in female humpback whales in the Northern Hemisphere [22,23,58,67,68]. For example, on the Hawaiian breeding grounds, Glockner-Ferrari & Ferrari [23] observed that four of 34 females experienced an annual reproductive cycle and one female was observed with different calves in four consecutive years. However, Glockner-Ferrari and Ferrari focused primarily on approaching mothers with a calf, leading to a likely ascertainment bias. Observations of annual calving are much lower on the feeding grounds. For example, Robbins [22] calculated that only 2% of females returned with calves in consecutive years in the Gulf of Maine.

### 4.4. Are observed rates of pregnancy ‘too high’?

In 1966, the IWC prohibited the commercial takes of humpback whales; this protection has now been in place for over 50 years [69]. At a global level, the recovery of humpback whale populations under this protection has been very effective, as reflected by a recent decision to down-list the species to Least Concern by the International Union for Conservation of Nature (2016), although a few populations in the Northern Hemisphere remain in poor conservation status. It is reasonable to infer that the high abundance of krill along the WAP [70] has supported the recovery of this feeding aggregation of humpback whales. Unfortunately, it is not possible to reconstruct the demographic trajectory of these whales over the last half-century because there is no good historical baseline for this population and we have just recently been able to assess demographic parameters, such as pregnancy rates, in these whales.

We believe that the existence of annual reproduction in this population represents a response to favourable ecological conditions, as humpback whales along the WAP recover from past over-exploitation. The frequent occurrence of an annual reproductive cycle, together with a high mean pregnancy rate of 63.5%, in female humpbacks suggests a population that is growing rapidly [23]. Indeed, the WAP feeding aggregation of humpbacks (and nearly all populations of this species) is increasing [60,71]. However, our pregnancy rates are much higher than expected for a population that is believed to be growing at a rate of only 3.4% per year [60]. Similarly high observations of annual reproduction and overall pregnancy rates were made among humpbacks sampled of the Kermadec Islands in Oceania [72]. The east Australian population of humpbacks is projected to be recovering at or near the estimated physiological limit of the species (approx. 10% per year) [73,74]. The differences between the high pregnancy rates we observed and the rather pedestrian rates of population growth estimated for breeding stock G could reflect one or more of the following: the estimate of population growth is biased low; pregnant females experience very high rates of perinatal loss, and or mortality of the calves on the breeding grounds; or there is significant spatial heterogeneity with respect to reproductive state on the foraging grounds.

The growth of a population depends not only on its reproductive rate, but on the survival and recruitment of calves into the population [75]. It is possible that the occurrence of an annual pregnancy cycle in this population results from the early mortality of a calf or late term fetus during the previous cycle [63,75]. There are no data on fetal loss in this species, but calf mortality is known to occur after birth but before arrival on the feeding grounds in some populations [76]. We believe that it is unlikely that significant fetal loss is occurring in humpback whales along the WAP, because the population is recovering from past depletion and because of a lack of other baleen whale populations having similarly recovered, it is likely that there is no competition for resources (e.g. food is not limiting) and whales can feed unrestricted. Resolution of this question will require more information on the reproductive histories of individual females and, particularly, on the fate of their dependent calves. Our future work, specifically genetic and photographic capture–recapture studies of mothers with calves, will focus on addressing this knowledge gap.

We sampled whales opportunistically, avoiding re-sampling an individual when possible. However, it is possible that the high rates of pregnancy we observed could be derived from some spatial artefact. Perhaps different demographic groups (e.g. lactating females, pregnant females) distribute themselves unevenly within the feeding area. We believe that this possibility is unlikely due to the fact that these animals range over very broad spatial scales during the feeding season [77]. However, future work is required to address this possibility.

## 5. Conclusion

Our research demonstrates that this feeding aggregation of humpbacks exhibits high pregnancy rates and a high proportion of females that are simultaneously pregnant and lactating. Both of these findings are consistent with a rapidly growing population, but are not supported by current estimates of population growth for the WAP population. Our work builds on the demographic and life-history data of Southern Hemisphere humpbacks derived from whales killed during the last century and published 50 years ago. Our intent here is to document the current demography of this population and establish a baseline with which to assess the impact of future climatic trends. As the extent of winter sea ice continues to decline, it is possible that we will witness a temporal shift and spatial expansion of the feeding grounds of these whales, at least in the short term, and prey availability will likely increase [78–80]. Long-term trends however, may be more problematic, due to the tight coupling of sea ice and the recruitment of krill in the WAP ecosystem [81,82]. We have already witnessed responses in the demography of other baleen whales to climate change [79,83]. We will continue to monitor this feeding aggregation of whales to document its recovery and response to future environmental changes.

## Supplementary Material

Electronic supplementary material table header page

## Supplementary Material

Table S1: Data associated with sample collection and genetic information

## Supplementary Material

Table S2: Data associated with blubber hormone evaluations for pregnancy state

## Supplementary Material

Figure S1

## Data accessibility

The datasets supporting this article have been uploaded as part of the electronic supplementary material.
