# Supplementary figures and images for: High pregnancy rates in humpback whales (Megaptera novaeangliae) around the Western Antarctic Peninsula, evidence of a rapidly growing population
Source: R Soc Open Sci. 2018 May 2;5(5):180017. doi: 10.1098/rsos.180017 (PMC5990787; doi:10.1098/rsos.180017)

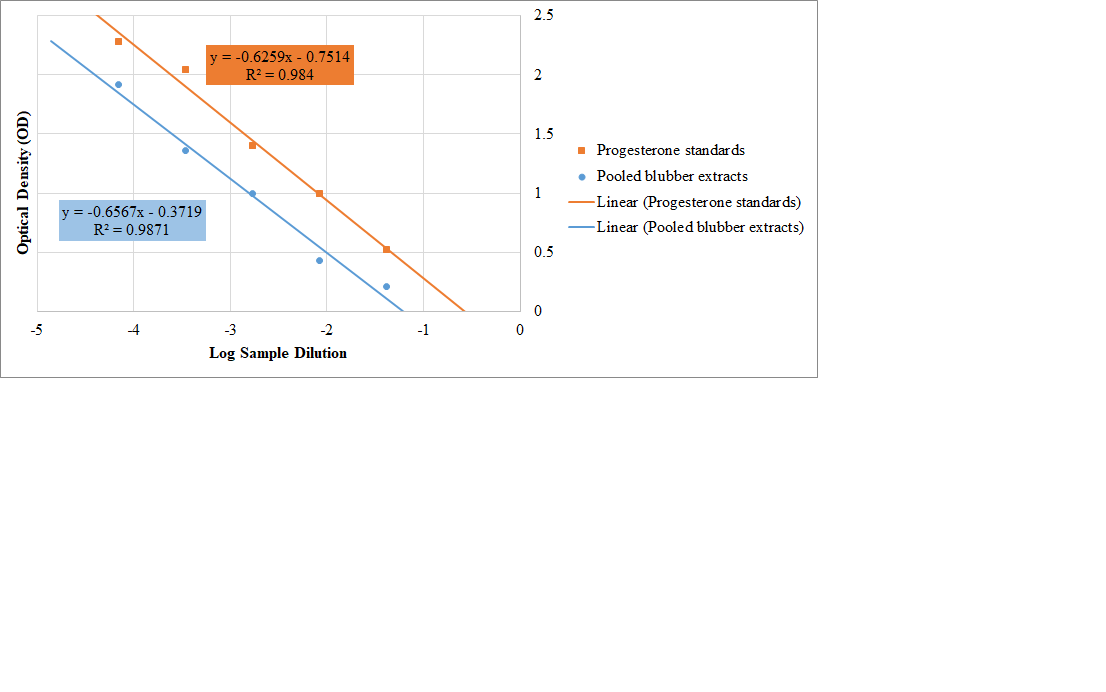

Supplement: Figure S1 [file rsos180017supp4.png]
